# Characterization of the biology and infectivity of *Leishmania infantum* viscerotropic and dermotropic strains isolated from HIV+ and HIV- patients in the murine model of visceral leishmaniasis

**DOI:** 10.1186/1756-3305-6-122

**Published:** 2013-04-26

**Authors:** Joana Cunha, Eugenia Carrillo, Carmen Sánchez, Israel Cruz, Javier Moreno, Anabela Cordeiro-da-Silva

**Affiliations:** 1 Parasite Disease Group, Unit of Infection and Immunity, IBMC - Instituto de Biologia Molecular e Celular, Universidade do Porto, Rua do Campo Alegre, 823, Porto, 4150-180, Portugal; 2 Instituto de Ciências Biomédicas Abel Salazar and Faculdade de Medicina, Universidade do Porto, Porto, Portugal; 3 WHO Collaborating Center for Leishmaniasis, Centro Nacional de Microbiologia, Instituto de Salud Carlos III, Madrid, Spain; 4 Laboratório de Microbiologia, Departamento de Ciências Biológicas, Faculdade de Farmácia, Universidade do Porto, Porto, Portugal

**Keywords:** *Leishmania infantum*, Clinical isolates, Visceral leishmaniasis, Molecular typing, Metacyclogenesis, Infectivity, Tropism

## Abstract

**Background:**

Leishmaniasis is a group of diseases with a variety of clinical manifestations. The form of the disease is highly dependent on the infective *Leishmania* species and the immunological status of the host. The infectivity of the parasite strain also plays an important role in the progression of the infection. The aim of this work is to understand the influence of the natural infectivity of *Leishmania* strains in the outcome of visceral leishmaniasis.

**Methods:**

In this study we have characterized four strains of *L. infantum* in terms of molecular typing, *in vitro* cultivation and differentiation. Two strains were isolated from HIV+ patients with visceral leishmaniasis (Bibiano and E390M), one strain was isolated from a cutaneous lesion in an immunocompetent patient (HL) and another internal reference strain causative of visceral leishmaniasis (ST) also from an immunocompetent patient was used for comparison. For this objective, we have compared their virulence by *in vitro* and *in vivo* infectivity in a murine model of visceral leishmaniasis.

**Results:**

Molecular typing unraveled a new *k26* sequence attributed to MON-284 zymodeme and allowed the generation of a molecular signature for the identification of each strain. *In vitro* cultivation enabled the production of promastigotes with comparable growth curves and metacyclogenesis development. The HL strain was the most infective, showing the highest parasite loads *in vitro* that were corroborated with the *in vivo* assays, 6 weeks post-infection in BALB/c mice. The two strains isolated from HIV+ patients, both belonging to two different zymodemes, revealed different kinetics of infection.

**Conclusion:**

Differences in *in* vitro and *in vivo* infectivity found in the murine model were then attributed to intrinsic characteristics of each strain. This work is supported by other studies that present the parasite’s inherent features as factors for the multiplicity of clinical manifestations and severity of leishmaniasis.

## Background

Parasites from the *Leishmania* genus are trypanosomatid protozoans responsible for a group of diseases with a broad range of clinical manifestations collectively known as leishmaniasis (reviewed in [1-3]). The emergence of leishmaniasis as an opportunistic infection in HIV+ patients in areas where both pathogens are endemic [4] has generated new interest in leishmaniasis.

It is well known that species such as *L. major* and *L. mexicana* are usually exclusively dermotropic, while *L. infantum* and *L. donovani* are responsible for both cutaneous and visceral leishmaniasis [5]. Apart from a general species-specific organ tropism of *Leishmania*, intraspecies intrinsic characteristics are also a relevant factor to consider. According to Maia *et al*. [6], dermotropic and viscerotropic *L. infantum* strains modulate the sand fly biting time on the host leading to the delivery, respectively, of a high or low dose of metacyclic promastigotes into the skin which will impact on the parasite tropism and manifestation of the disease. Even strains belonging to the same zymodeme have been associated to differential infectivity [7].

In experimental infections, however, another parasite-related feature is of major importance. *In vitro* cultivation of *Leishmania* is a subject open to wide variation between laboratories, making the comparison of similar experiments ambiguous. Depending on the culture medium (Santarém, N. and Cunha, J., submitted results and [8]), the duration of the culture [9] and the number of axenic passages performed [9], the promastigotes generated will be differentially enriched in metacyclic forms [9], which will condition the success of the infection. Nonetheless, the genetics and the immune status of the host play a similarly important role in the tropism and severity of the disease [10]. In the murine models, *L. major* was only found in the infection site of the resistant C57BL/6 mice after subcutaneous injection, whereas the same experimental protocol followed in the susceptible BALB/c strain allowed visceralization [11]. Also, high and low infective strains maintained their profile (visceralizing or regulatory, respectively) in BALB/c and C.B-17 SCID mice, although with higher parasite loads in the T and B cell-dysfunctional SCID animals [12].

The analysis of HIV/*Leishmania*-coinfected human patients brought important insights into the role of the immune system on the severity of the disease. On the one hand the visceralization of dermotropic strains is frequently observed in HIV/*Leishmania*-coinfections [13], as well as the regular presence of amastigotes in uncommon locations such as the lungs or the intestine [14]. On the other, the appearance of unique *Leishmania* zymodemes in HIV+ patients has been reported, which may be indicative of circulating strains normally associated with asymptomatic disease in immunocompetent patients [13,15]. Some studies have shown that strains originating from HIV+ patients have low infectivity, which explains its appearance only in immunocompromised individuals [7,16]. On the contrary, three distinct infective profiles were attributed to strains responsible for CL or VL (from immunocompetent or HIV+ patients) and no correlation was made according to the origin of the isolate [17].

In this study, we have focused on four different *L. infantum* strains isolated from patients with CL, VL and HIV/*Leishmania* coinfections. We characterized these strains according to molecular, biological and infectivity characteristics. We standardized the *in vitro* culture to avoid any biased infectivity that was evaluated with macrophage and mouse models. We have studied the distribution of the strains in acute and chronic infection by qPCR assessing the parasite load in spleen, liver, bone marrow, blood and lymph nodes and correlated differences in infectivity with major findings on the molecular typing.

## Methods

### Parasites

Four *L. infantum* strains isolated from patients in the Mediterranean basin and Portugal were used in this study. MHOM/MA/67/ITMAP-263 (ST) is a cloned line derived from a patient with visceral leishmaniasis [9,18] that was used as an internal and comparative control in all the experiments performed. HL strain (MHOM/PT/2009/LLM-1708) was isolated from an immunocompetent patient with cutaneous leishmaniasis. Briefly, a skin biopsy was dissociated in a cell strainer to isolate the cells and was then transferred into culture in RPMI. E390M (MHOM/ES/99/LLM-855) and Bibiano (MHOM/ES/01/LLM-1083) were isolated from bone marrow aspirates of HIV/*Leishmania*-coinfected patients, this second one being responsible for recurrent relapses of leishmaniasis. The bone marrow samples were cultivated in NNN medium at 26–27°C, until the expansion of the promastigotes. ST, HL and Bibiano strains have been characterized by multilocus enzyme electrophoresis (MLEE) as MON-1 zymodeme, while E390M is MON-284 (electrophoretic mobilities for malate dehydrogenase (MDH) and glucose-phosphate isomerase (GPI) were determined to be of 104 and 105, respectively, in relation to MON-1 zymodeme [13]).

### Molecular typing

*L. infantum* isolates were subjected to molecular typing by targeting four different regions of the *Leishmania* genome. *Leishmania* DNA was extracted by phenol/chloroform as described below in more detail and samples were adjusted to a final concentration of 10 ng/μL after measuring DNA content with a Nanodrop ND-1000 spectrophotometer (Thermo Scientific). A volume of 5 μL of each sample was used in further PCRs. PCR products were run on 2% agarose gels stained with ethidium bromide and visualized under UV light. Then they were excised from agarose gels and purified using the QIAquick Gel Extraction Kit (QIAGEN). First, the species status of the isolates was confirmed by DNA sequencing of the heat-shock protein 70 (*hsp70*) gene [19]. Further subtyping was performed by sequence analysis of the ribosomal internal transcribed spacer 1 (ITS1) and 2 (ITS2) [20] and the hydrophilic acylated surface protein B (*hasp*B) or *k26* gene [21]. The Big-Dye Terminator Cycle Sequencing Ready Reaction Kit V3.1 and the automated ABI PRISM 377 DNA sequencer (Applied Biosystems) were used for direct sequencing of the *k26*, ITS1 and ITS2 PCR products that was performed with the corresponding forward and reverse primers; internal primers for sequencing were also used for the *hsp70* PCR product, as described by Fraga *et al*. [19]. The obtained sequences were analyzed and edited using the software BioEdit Sequence Alignment Editor, version 7.0.9.0 (Ibis Biosciences) [22]. ClustalW multiple alignment algorithm tool and manual adjustment were used for comparison of the resulting sequences with the respective published sequences. The *hsp70* sequences were compared with those of the different *Leishmania* species generated by Fraga *et al*. [19]. ITS types were assigned to each isolate according to the sequence polymorphism of the 12 microsatellite regions included in ITS1 (four sites) and ITS2 (eight sites), as described by Kuhls *et al*. [20]. *k26* genotypes were assigned according to the size and sequence of the PCR product, following the criteria previously described by Haralambous *et al*. [21].

For the generation of unique patterns that could be used for strain identification, we amplified a region of the kinetoplast DNA minicircles and evaluated the restriction profile after *Hae*III (Roche Applied Science) endonuclease digestion [23] using DNA extracted from axenic promastigotes and from experimentally infected murine tissues.

### Culture media

Novy-MacNeal-Nicolle medium (NNN) was prepared with a semi-solid phase made of 1.4% agar (Sigma-Aldrich), 0.6% NaCl (Merck), 31% defibrinated rabbit blood, 625 units/mL penicillin, 625 units/mL streptomycin and RPMI 1640 medium supplemented with 10% fetal bovine serum (FBS), 2 mM L-glutamine, 100 U/mL penicillin, 100 U/mL streptomycin and 20 mM HEPES buffer (all from Lonza) as liquid phase.

### Growth curves and viability

Parasites were first passed in mice to control their virulence and frozen in vials for future use until 10 *in vitro* passages [9]. Promastigotes were cultivated at 26°C with an initial inoculum of 10^6^ parasites/mL in NNN from a synchronized culture in the same media and followed for 6 days. In each day, parasites were counted in a hemocytometer and stained with Annexin V and 7-amino-actinomycin D (7-AAD) for viability analysis as described in [9]. 10 000 gated events were analyzed in a FACSCanto II (BD Biosciences) and the percentage of Annexin^-^/7AAD^-^ cells determined with FlowJo software (TreeStar).

### Cell cycle

In each day of culture, promastigotes were recovered and washed in PBS/FBS 2%. 2×10^6^ parasites were resuspended in 1 mL of PBS/FBS 2% and 3 mL of ice-cold absolute ethanol (Panreac) was carefully added while vortexing. After fixation for 1 hour at 4°C, the parasites were washed in PBS and resuspended in 1 mL of propidium iodide (PI) staining solution consisting of citrate buffer 3.8 mM in PBS, 50 μg/mL PI (Sigma-Aldrich) and 0.5 μg/μL RNAse A (Sigma-Aldrich). Following an incubation of 30 minutes at 4°C, 20 000 single live cells were acquired in a FACSCanto II and analyzed with the FlowJo’s cell cycle built-in tool.

### Quantification of metacyclogenesis-dependent gene transcription

10^7^ promastigotes from day 1 to 6 of culture were resuspended in TRIzol reagent (Invitrogen) and frozen at −80°C. Total RNA was extracted using chloroform and isopropanol according to the manufacturer’s instructions and solubilized in 10 μL of nuclease-free water. RNA of high quality was obtained (RQI between 9.0 and 10.0) as assessed using RNA StdSens Chips of the Experion automated electrophoresis system (Bio-Rad). RNA concentration was determined using a Nanodrop ND-1000 spectrophotometer. Samples were stored at −80°C until cDNA was synthesised. Reverse transcription was performed with iScript cDNA synthesis kit (Bio-Rad) according to the manufacturer’s instructions over 500 ng of total RNA. *Meta-1*, Small Hydrophilic Endoplasmic Reticulum-associated Protein (*SHERP*) and *histone H4* transcription was quantitatively analyzed after normalization with *rRNA45* transcription by qPCR using the iQ SYBR Green Supermix according to the manufacturer’s instructions in a My iCycler iQ5 (Bio-Rad). 4 μL of cDNA (diluted 25×) was used as template that was run in duplicate with 500 nM (*Meta-1* and *histone H4*) or 250 nM (*SHERP* and *rRNA45*) of the following primers (from Stabvida): *Meta-1* [GenBank: NC_009401] forward: 5′-GGGCAGCGACGACCTGAT-3′ and reverse: 5′-CGTCAACTTGCCGCCGTC-3′ (modified from [24]); *histone H4* [LinJ35.1400, GenBank: XM_001468907] forward: 5′-ACACCGAGTATGCG-3′ and reverse: 5′-TAGCCGTAGAGGATG-3′ [9]; *SHERP* [GenBank: XM_003392466] forward: 5′-CAATGCGCACAACAAGATCCAG-3′ and reverse: 5′-TACGAGCCGCCGCTTATCTTGTC-3′ [9]; *rRNA45* [GenBank: CC144545] forward: 5′-CCTACCATGCCGTGTCCTTCTA-3′ and reverse: 5′-AACGACCCCTGCAGCAATAC-3′ [25]. Changes in relative gene expression were determined with ΔΔCT method and results show fold changes comparative to day 1 calculated by 2^-ΔΔCT^.

### *In vitro* infections

Bone marrow-derived macrophages (BMMo) were produced as described previously [9]. Stationary promastigotes cultivated in NNN for 4 days were washed and put in contact with the cells in 1:10 ratio (cell:parasites) for 4 hours. Extracellular parasites were washed away with PBS and the cells incubated for more 24, 48, 72 or 96 hours or fixed immediately with 2% PFA. The macrophages were mounted on Vectashield with DAPI (Vector Laboratories) and 100 infected cells or 400 total cells were counted in duplicate by fluorescence microscopy in a Zeiss Axioskop (Carl Zeiss). The percentage of infected cells and the geometric mean of the number of parasites per infected cell were evaluated. The infection index was calculated by multiplication of both parameters to account for the overall parasite load.

### *In vivo* infections

7–8 week-old BALB/c male mice (4–5 animals per group, except in 2-week infections with E390M strain where only 3 animals were used) were infected via the intraperitoneal route with 10^8^ promastigotes of each strain cultivated in NNN for four days. After 2 or 6 weeks of infection mice were anesthetized with isoflurane and sacrificed by cervical dislocation. Blood, inguinal lymph nodes, spleen, liver and femoral bone marrow were recovered for quantification of parasite load. Blood and spleen were also used for the evaluation of humoral and cellular responses.

### Parasite load quantification

Parasite load was quantified in samples that were collected and frozen at the time of animal sacrifice. We used 200 μL of blood collected with EDTA, 10 mg of spleen and liver (single cell suspensions), 3 × 10^6^ bone marrow cells and the inguinal draining lymph node to extract DNA. First, 400 μL of a buffer containing 10 mM NaCl, 10 mM EDTA and 10 mM Tris–HCl with pH 8.0 were added to the samples, which were incubated overnight with 40 μg of proteinase K (Sigma-Aldrich) at 56°C with shaking. Then, the samples were vortexed and incubated for 20 minutes at 70°C. DNA was extracted using phenol/chloroform/isoamyl alcohol (all from Merck Millipore). After precipitation with ice-cold 70% ethanol solution, DNA was dissolved in 100–200 μL of nuclease-free water. We quantified the total DNA in a Nanodrop ND-1000 spectrophotometer and prepared dilutions of concentrations adjusted for each tissue. We quantified *Leishmania sp*. DNA by qPCR using 1000 nM of R223 and 500 nM of R333 primers (Sigma-Aldrich) for the small subunit rRNA (SSUrRNA) [26]. Depending on the tissue, 100 to 400 ng of total DNA served as a template in a 20 μL reaction using LightCycler FastStart DNA Master SYBR Green I kit (Roche Applied Science) according to the manufacturer’s instructions, in a touchdown qPCR performed in a LightCycler 2.0 carousel-based instrument (Roche Applied Science) with final annealing temperature of 65°C [27]. CTs were extrapolated in a standard curve constructed with serial dilutions of *L. infantum* DNA (strain JPC, MCAN/ES/98/LLM-722) diluted in host DNA (from spleen of naïve mice) to calculate *Leishmania* content in parasites/μg DNA. Whenever the qPCR gave a positive (with the expected melting curve) but unquantifiable value or a doubtable specific product (aberrant melting curve), we performed a nested PCR [28] that has a higher sensitivity (0.01 parasites) than the qPCR (0.6 parasites) to confirm the positivity of the quantitative result. 300 nM of R221 and R332 primers [26] were used for the first amplification reaction. For the second reaction, 10 μL of the first PCR product diluted 1:40 served as template with the same R223 and R333 primers (300 nM and 150 nM, respectively) used for the qPCR. This molecular quantification was applied after proper validation by comparison with limiting dilution assay (Additional file 1: Additional Methods and Additional file 2: Figure S1).

### Splenic cell populations

5 × 10^5^ splenocytes were surface-stained for 20 minutes at 4°C with saturating concentrations of monoclonal antibodies (all from Biolegend). After washing twice with PBS/FBS 2%, the cells were examined by flow cytometry in a FACSCanto (BD Bioscences) and analyzed with FlowJo software. After acquisition of 50000 cells identified by FSC and SSC parameters, major populations were identified as follows: CD4^+^ T lymphocytes (PerCp.Cy5.5 anti-CD3, clone 17A2; APC.Cy7 anti-CD4, clone GK 1.5), CD8^+^ T lymphocytes (PerCp.Cy5.5 anti-CD3; FITC anti-CD8, clone 53–6.7), B cells (FITC anti-CD19, clone 6D5), monocytes/macrophages (PE.Cy7 anti-CD11b, clone M1/70; PerCp.Cy5.5 anti-Ly6C, clone HK1.4).

### *Leishmania*-specific immunoglobulins

The specific humoral response was analyzed by ELISA as described elsewhere [18]. In short, 96-well microtitration plates (Greiner Bio-One) were coated with 10 μg/mL of soluble *Leishmania* antigens (SLA) in carbonates buffer pH 8.5 and then blocked with PBS/gelatin 1%. Sera were diluted 1:100 and incubated for 2 hours at 37°C. After washing with PBS/tween 20 0.1%, HRP-conjugated anti-IgG1 or anti-IgG2a (Southern Biotech) were added to the wells at a dilution of 1:5000 and incubated for 30 minutes at 37°C. The plates were revealed with 0.5 mg/mL of o-phenylenediamine dihydrochloride (Sigma-Aldrich) in citrate buffer pH 4.0 and the reaction was stopped with HCl 3 N. The absorbance was read at 492 nm in a Synergy 2 microplate reader (Biotek).

### Animals and ethics statement

For the *in vitro* experiments we used 10–12 week-old female BALB/c mice bred and maintained at IBMC - Instituto de Biologia Molecular e Celular (Portugal) animal facilities. For the *in vivo* experiments 7–8 week-old male BALB/c mice were bred and maintained at the Instituto de Salud Carlos III (Spain) animal facilities. Mice were housed in IVC cabinets with sterile food and water *ad libitum*. All experiments conducted were carried out in accordance with the IBMC.INEB and ISCIII Animal Ethics Committees and the Portuguese and Spanish National Authorities for Animal Health guidelines that follow the statements on the directive 2010/63/EU of the European Parliament and of the Council. ACS has an accreditation for animal research given from Portuguese Veterinary Direction (Ministerial Directive 1005/92).

### Statistical analysis

GraphPad Prism 5 (GraphPad Software) was used to perform all the statistical analysis. The results are presented as means ± standard deviations (SD). To compare statistical differences between means two-sided *t* test or one-way ANOVA followed by Dunnett’s multiple comparison test were run when comparing 2 or more groups, respectively, unless otherwise stated. * p < 0.05, ** p < 0.01 and *** p < 0.001.

## Results and discussion

### Molecular characterization of the clinical isolates of *L. infantum*

To understand the intraspecies polymorphisms and its possible impact on both *in vitro* and *in vivo* infectivity, we characterized certain molecular aspects of these four *L. infantum* strains.

**Figure 1:**
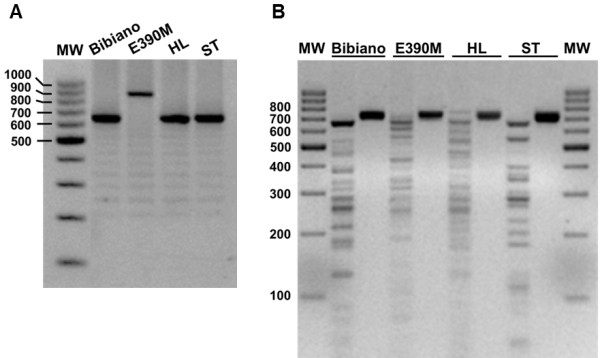
**Molecular characterization of *L. infantum *isolates. (A)** Amplification of the *k26* gene. See Additional file 3: Figure S2 for coding DNA sequences and alignment. **(B)** RFLP patterns for *L. infantum* isolates after *Hae*III digestion of an amplified fragment of the minicircles kDNA. Profiles for DNA extracted from axenic promastigotes are shown, though DNA from experimentally infected murine tissues delivered comparable restriction patterns. For each strain, left lane corresponds to the digested DNA and right lane to undigested product. Gels were prepared with 2% (**A**) or 2.5% (**B**) agarose and stained with ethidium bromide. MW - molecular weight marker with bp units assigned in the left of the figure.

A molecular approach was followed by us aiming not only to confirm the identity [19] of Bibiano, E390M and HL strains, together with the laboratory’s standard *L. infantum* (ST) strain, but also to subtype the isolates according to Haralambous *et al.*[21] and Kuhls *et al.*[20]. Molecular genotyping of the strains indicated that all four were 100% consistent with *L. infantum* and were clustered in the same ITS type A group (data not shown), which is the most common in specimens from the Mediterranean area even within different zymodemes [20]. Moreover, Bibiano, HL and ST were classified in the *k26* group 1b, the most frequent in the Iberian Peninsula [21], while E390M belongs to a new *k26* group reported here for the first time, since it returned an 836 bp amplicon [GenBank: KC576808] never found before (Figure 1A and Additional file 3: Figure S2).

PCR-RFLP of kinetoplast DNA minicircles was used as a tool for creating an individual identity for each strain. Because we were working with *L. infantum* strains that preferably could have similar growth and morphology, they would be indistinguishable in *in vitro* cultures. In case of cross-contaminations between strains [29,30], we would like to have a tool for identification of our parasites. All the strains showed very complex profiles (Figure 1B), but each one of the specimens was clearly identifiable by the examination of the most intense bands.

### Characterization of biological features of the promastigotes generated *in vitro*

*In vitro* cultivation has a major impact on the virulence of the pathogens due to intrinsic properties of the culture media that modulate *Leishmania* infectivity (Santarém, N. and Cunha, J., submitted results and [8,31]), or to the loss of adaptive capacities to mammalian host cells resulting from long-term *in vitro* cultivation of promastigotes. A relevant experimental bias can be introduced if these factors are not considered. Hence, the comparison of the infective capacity of distinct strains should take into account the adaptation to culture conditions and/or the axenic growth behavior [7,32].

**Figure 2:**
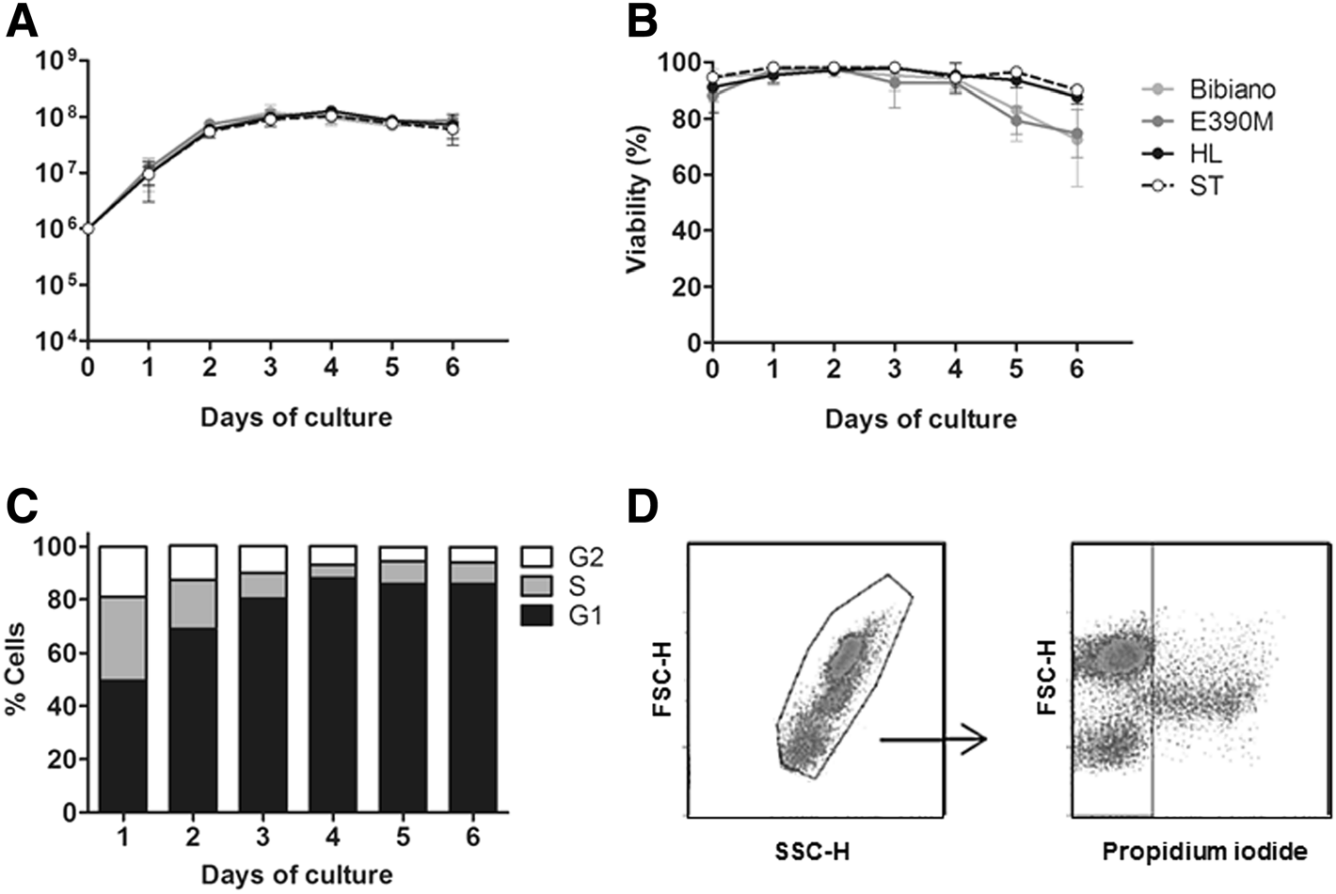
***In vitro *development of *L. infantum *strains. (A)** Growth curves of Bibiano (light grey), E390M (dark grey), HL (black) and ST (white) *L. infantum* strains in NNN. Cultures were started with 10^6^ promastigotes of each strain per mL and growth was followed for a week, with a daily counting of parasite numbers in a Neubauer chamber. **(B)** Parasite viability measured by flow cytometry as percentage of AnnV^-^/7AAD^-^ cells. The mean of three independent experiments is plotted; bars represent SD. **(C)** Cell cycle analysis of HL during growth period. Data show means of one representative experiment of three independent assays. **(D)** Illustrative phenotype of *L. infantum* promastigotes with 4 days of culture in NNN captured by flow cytometry.

We started to characterize the axenic growth of the four *L. infantum* strains. Aiming to identify any difference in their infectivity that could justify the dissimilar outcomes of the disease in the natural infections, we first had to understand what specific nutritional needs the parasites might have in order to standardize the *in vitro* culture conditions. Preliminary experiments with parasites cultivated in RPMI medium exposed remarkable differences among the growth of each *L. infantum* strain (data not shown). Attempts to find a culture medium suitable for the continuous and comparable growth of the four strains included doubling the FBS content to 20% and adding glucose to RPMI medium, the use of Schneider’s Insect medium and the preparation of a mixture of RPMI and Schneider media in equal parts (data not shown; the composition of these culture media is detailed in Additional file 1: Additional Methods). However, only the use of Novy-MacNeal-Nicolle medium (NNN) allowed us to guarantee an *in vitro* homogeneous culture condition where all four strains could be easily cultivated, maintained and have a similar development that would allow the direct comparison of strain infectivity. Bibiano, E390M, HL and ST strains showed perfectly overlapped growth curves in NNN (Figure 2A), with morphologically indistinguishable promastigotes that maintained high viability (90-95%) at least until the fourth day of culture (Figure 2B).

**Figure 3:**
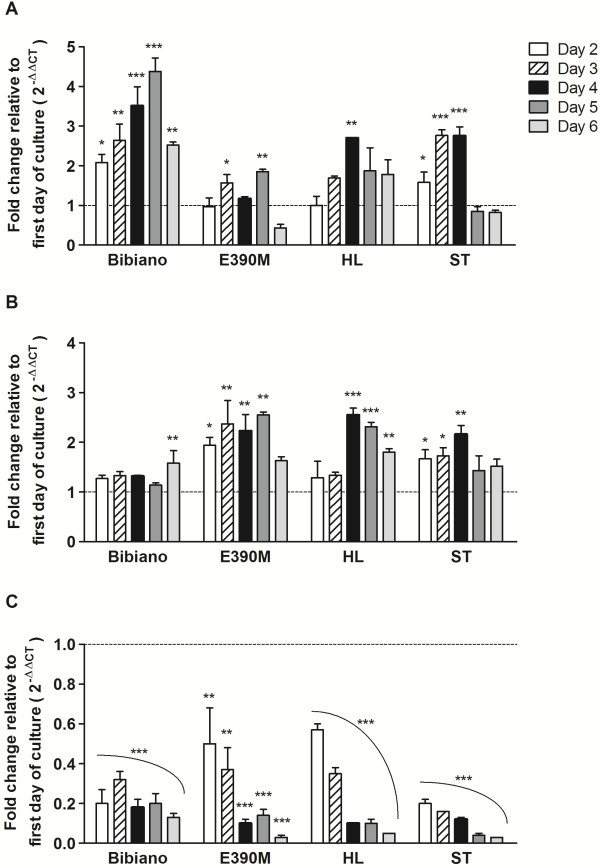
**Indirect measurement of metacyclogenesis.** Quantification of **(A)***Meta-1*, **(B)***SHERP* and **(C)***histone H4* transcription by RT-PCR over the time of culture in NNN medium for the four strains. Bars represent the mean fold change relative to day 1 with SD of two independent experiments. Statistical significant differences between day 1 and the following days were determined with One-way ANOVA and Dunnett’s multiple comparison test.

The culture of all promastigote strains in NNN enabled a rapid exponential parasite growth that peaked at day 3 (≈10^8^ parasites/mL) after which it stabilized until day 6. These timings could be confirmed by the analysis of the cell cycle (Figure 2C and Additional file 4: Figure S3). On the first day of culture, around 50% of the parasites were in S/G2 phases in a clear indication of active proliferation. From the third day on, only ≈ 20% of the cells were found to be in division. Moreover, during the stationary phase promastigotes with small bodies and large flagella could be clearly identified by optic microscopy (data not shown); these were confirmed by flow cytometry (Figure 2D) as FSC^lo^PI^-^ cells [33,34]. Based on this typical morphology of metacyclic promastigotes, we analyzed metacyclogenesis throughout the 6 days of culture by quantifying the expression of specific genes which are upregulated (*Meta-1* and *SHERP*) or downregulated (*histone H4*) during the process [25,33,35] (Figure 3 and Additional file 5: Figure S4). Despite the observed interstrain variations on the fold modifications of each gene, all of the strains showed a dramatic decrease in *histone H4* expression consistent with the cell cycle analysis. For Bibiano, *Meta-1* gene expression significantly increased every day of culture, while for E390M the major fold changes were detected in the *SHERP* gene expression. Both *Meta-1* and *SHERP* increased overtime for HL and ST until day 4 and after that they recovered to levels close to those of day 1. We cannot directly compare the metacyclogenesis process between the four strains, but we must take into account the total information available to affirm that these promastigotes were differentiating into metacyclic forms. The leading factor is that the overall analysis of the three genes studied pointed towards metacyclogenesis. This trend could be better understood when calculating the ratio between the up and downregulated genes (Additional file 5: Figure S4), as all the strains presented an increase in the ratios over the 6 days of culture.

### Evaluation of the infectivity of *L. infantum* isolates

**Figure 4:**
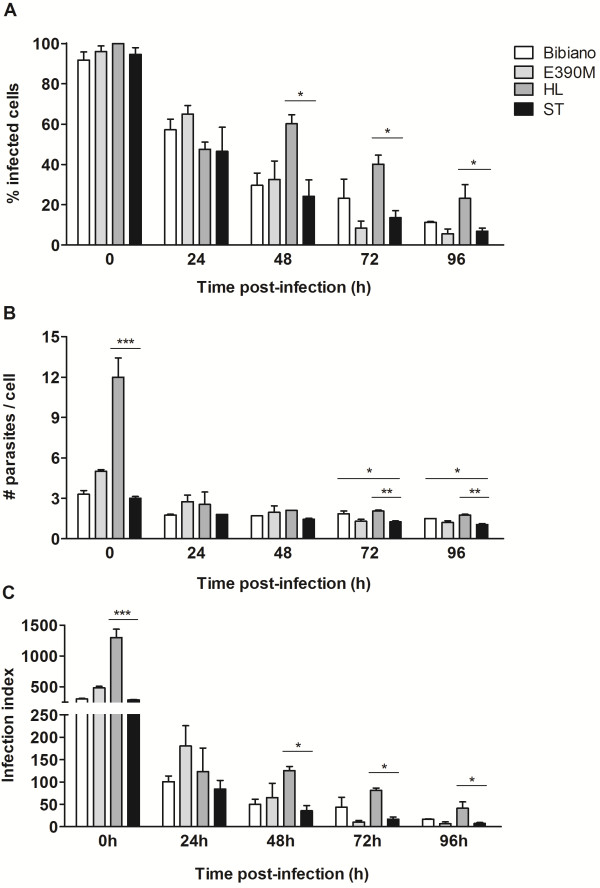
***In vitro *differential infectivity of *L. infantum *strains.** BMMo were infected with promastigotes of each strain after 4 days of culture in NNN in a ratio of 10:1 (parasites:macrophage). The kinetics of infection was followed counting **(A)** the infected cells and **(B)** the number of parasites per infected cell on fluorescent microscope. To account to the overall parasite load, an infection index **(C)** was calculated multiplying the individual data from (**A**) by (**B**). Statistically significant differences between ST and the other strains were determined with One-way ANOVA followed by Tukey’s multiple comparison test.

To evaluate parasite infectivity, we infected macrophages and followed the progression of the infection for four days (Figure 4). At the initial time-point, the four strains infected approximately all the cells without any differences in the total percentage of infected macrophages (Figure 4A). However, when evaluating the parasite load in each infected cell, HL promastigotes were more effective in the invasion of the macrophages with a mean of 12 ± 1.5 parasites in each infected cell versus 3 to 5 parasites found for the other strains (Figure 4B). After the first 24 hours, an accentuated reduction in the infection index was detected for the four strains (Figure 4C), which was maintained, though softened, during the entire period of the assay. Nevertheless, HL parasites were more resistant to macrophage-specific killing machinery as displayed by the increased infection index throughout the study. For this reason, this strain was considered to be more infective than the ST virulent strain [36,37]. Interestingly, 72 and 96 hours post-infection, macrophages could harbor significantly more Bibiano than ST parasites, though it did not translate into a higher overall parasite load as shown for HL (Figure 4C) because of the low percentage of infected cells.

**Figure 5:**
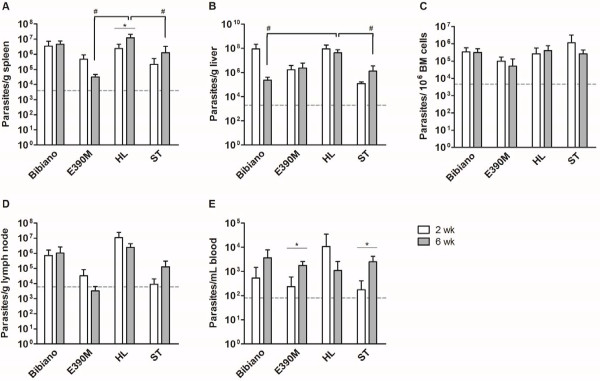
**Quantitative distribution of *L. infantum *in BALB/c mice 2 and 6 weeks after infection.** The parasite burden was quantified on the **(A)** spleen, **(B)** liver, **(C)** bone marrow, **(D)** lymph node and **(E)** blood. Data represent means ± SD of 3–5 animals per group of a representative experiment of two independent assays. *T* test was used to determine statistically significant differences between 2 and 6 weeks of infection with each strain; * p < 0.05. With one-way ANOVA followed by Tukey’s multiple comparison test we calculated statistically significant differences between strains at each time point; # p < 0.05. Dashed line indicate limit of detection for quantification for each tissue.

As is well known, disease manifestation depends on the virulence of *Leishmania* strain [12,17] but also on the genetic background [38] and immune status of the host [14,39]. Hence, the differences in infectivity detected *in vitro* were explored in the BALB/c model of visceral leishmaniasis to evaluate the influence of the parasites’ intrinsic characteristics in the ability to cause the disease. We therefore studied parasitological and immunological features in the acute and chronic phases of infection. Parasite load was quantified in the spleen, liver and bone marrow as the main target organs in this model. The presence of *Leishmania* in the draining lymph nodes and the blood were also investigated (Figure 5).

In the acute phase, 2 weeks after the infection, no significant differences were calculated between the four strains in all the five tissues studied. Despite the lack of statistical significance, important differences could be appreciated between strains. In the visceral organs (Figures 5A and B), HL and Bibiano showed very similar parasite burden, representing the most infective strains in these tissues. As compared to ST, they presented over 1 logarithm higher parasite load in spleen and were ≈ 800 times higher in the liver. ST was indeed the least efficient strain colonizing these organs, as E390M was able to infect almost 3 and 15 times more in the spleen and liver of mice. In the inguinal lymph nodes (Figure 5D) and the blood (Figure 5E), HL was the strain that showed higher parasitism. Its values surmounted 14, 300 and 1100 times the burden of Bibiano, E390M and ST, respectively, in the lymph nodes, and 3, 20 and 46 times, respectively, in the blood. The bone marrow was more infected by ST, though without accentuated differences between the other strains (Figure 5C). After this analysis one could expect ST to be the least infective strain in the acute phase of the disease, once HL was the strain that showed higher infectivity in the majority of the tissues examined, followed close by Bibiano. Nonetheless, estimating the total number of parasites existing in the whole animal, we calculated ≈ 5 × 10^8^ ST parasites, approximately double the number estimated for Bibiano and HL and over a logarithm more than E390M (Table 1).

**Table 1:** **Estimated overall parasite load of *L. infantum*-infected BALB/c mice 2 and 6 weeks post-infection**

**Total parasite load**	**2 weeks post-infection**				**6 weeks post-infection**			
	**Bibiano**	**E390M**	**HL**	**ST**	**Bibiano**	**E390M**	**HL**	**ST**
Spleen	67.7 × 10^4^	8.82 × 10^4^	44.1 × 10^4^	3.81 × 10^4^	62.7 × 10^4^	0.40 × 10^4^	329 × 10^4^	18.9 × 10^4^
Liver	157 × 10^6^	2.77 × 10^6^	112 × 10^6^	0.14 × 10^6^	0.35 × 10^6^	2.54 × 10^6^	86.8 × 10^6^	2.42 × 10^6^
Bone marrow	15.0 × 10^7^	4.49 × 10^7^	11.9 × 10^7^	50.9 × 10^7^	13.9 × 10^7^	2.29 × 10^7^	18.1 × 10^7^	11.7 × 10^7^
Lymph nodes	4.83 × 10^4^	0.19 × 10^4^	67.0 × 10^4^	0.10 × 10^4^	1.78 × 10^4^	0.015 × 10^4^	10.3 × 10^4^	0.59 × 10^4^
Blood	0.97 × 10^3^	0.42 × 10^3^	19.4 × 10^3^	0.32 × 10^3^	6.54 × 10^3^	3.12 × 10^3^	1.95 × 10^3^	4.53 × 10^3^
Whole animal	3.08 × 10^8^	0.48 × 10^8^	2.32 × 10^8^	5.09 × 10^8^	1.40 × 10^8^	0.25 × 10^8^	2.71 × 10^8^	1.19 × 10^8^

Mean weights of spleen, liver and inguinal lymph nodes of the infected animals (Additional file 6: Figure S5) were used in these calculations. Values for total volume of blood (1.8 mL) and total number of bone marrow cells (4.5 × 10^8^) were estimated as published elsewhere [40,41].

In the chronic phase, 6 weeks after infection, the four strains showed relative infection profiles in the spleen (Figure 5A), bone marrow (Figure 5C) and lymph nodes (Figure 5D) similar to the acute phase. As before, HL was shown to be the most infective strain in spleen and lymph nodes. In the bone marrow the differences were once more not as accentuated between strains, although the higher parasite loads were found in HL infected mice. In the liver (Figure 5B), HL was still the most infective strain, but Bibiano, which in the other tissues presented comparable parasite loads, was, significantly, the least infective strain, whereas E390M and ST produced intermediate infections. In the blood (Figure 5E), the differences observed at 2 weeks post-infection were neutralized, as the four strains showed similar levels of circulating parasites.

### Distribution and compartmentalization throughout the infection

Evaluating the progression of the disease, these four strains depicted very different trends. In the acute phase of infection, Bibiano and HL were found in very high numbers in the visceral organs but evolved in the opposite directions with chronicity. Bibiano was efficiently cleared from the liver, with a 425-fold reduction, though in the spleen the parasite load did not alter. As to HL, the liver infection was maintained and the splenic parasite burden increased 5 times from 2 to 6 weeks after infection, which showed not only high capacity to infect but also to perpetuate in the host. E390M, on the contrary, showed a low infective phenotype, with the lowest parasite loads in all the tissues quantified in the acute phase of infection. Through time, this strain was not able to proliferate in the spleen or in the bone marrow; the parasites resisted in the liver and showed a 7.4-fold increase in the blood. Despite with disparate initial parasite loads (more than 6-fold difference), Bibiano and E390M, both isolated from HIV+ patients, followed a very similar trajectory in the progression of the disease and, eventually, would be eliminated over time in these immunocompetent BALB/c mice. The high parasitemia presented by these two strains in the chronic phase may facilitate the anthroponotic transmission of *Leishmania* between the intravenous drug users (IVDUs) [42], one of the populations with highest risk of HIV/*Leishmania* coinfection [43]. Concerning ST, the standard virulent strain in our laboratory, showed a clear tropism for the bone marrow in the acute phase, with the lowest parasite loads in the remaining organs compared to other strains. However, 6 weeks post-infection, ST dramatically multiplied reaching levels ≈ 6- and ≈ 17-fold higher in the spleen and liver, respectively.

As a final remark, this study allowed us to verify that bone marrow parasite load is maintained in a range that does not suffer major alterations either over-time nor is it strain-dependent. Possibly this is the reason why bone marrow aspirates are the eligible sample for leishmaniasis diagnosis, either for microscopic analysis, culture or molecular techniques [4].

### Infectivity relates to cell modulation

**Figure 6:**
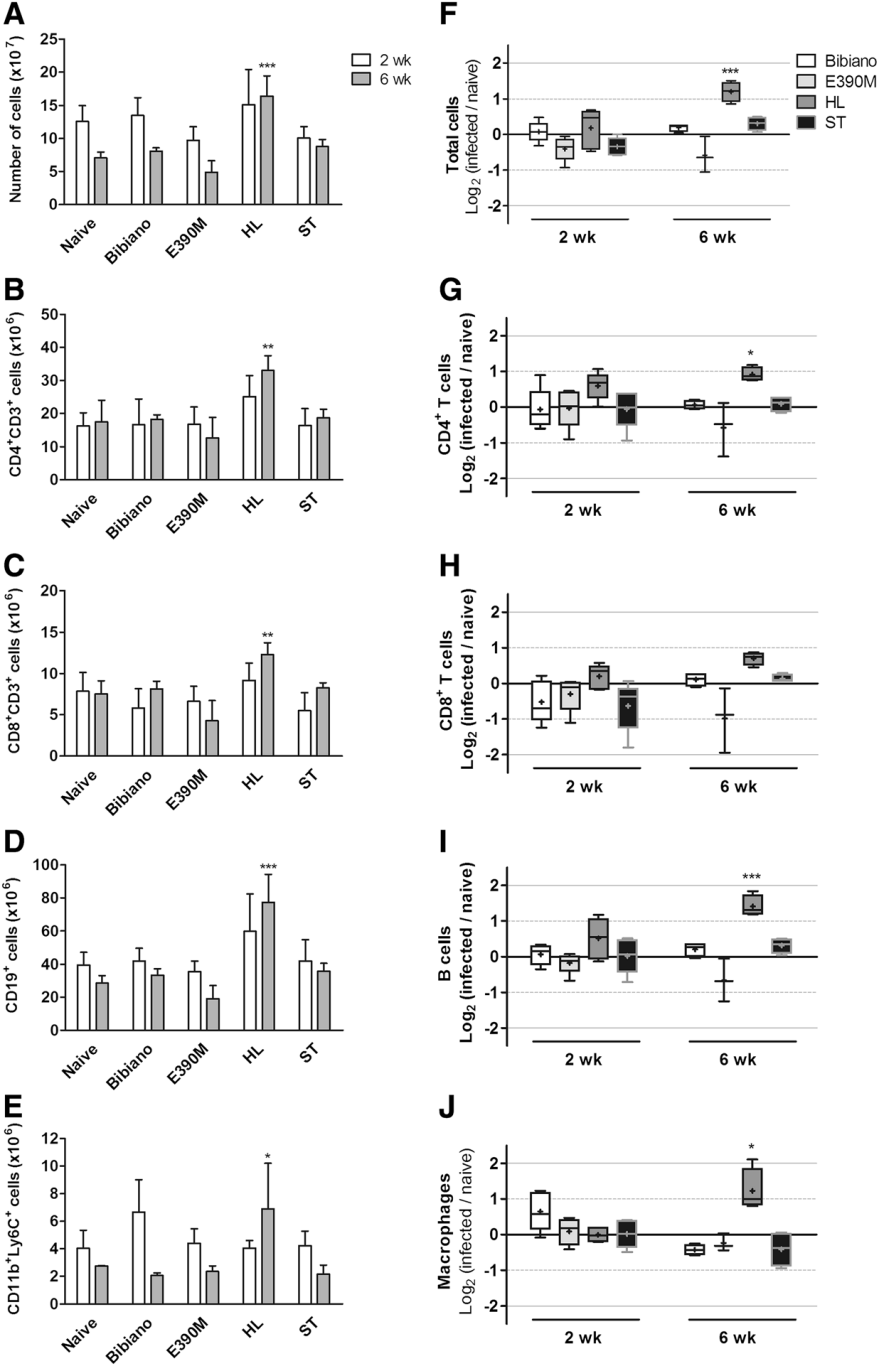
**Cell populations in spleens of naive and *Leishmania*-infected mice in the acute and chronic phases. (A), (F)** Total cells were counted and stained for identification of major splenic populations by flow cytometry. **(B), (G)** CD4^+^ T cells. **(C), (H)** CD8^+^ T cells. **(D), (I)** B cells. **(E), (J)** Monocytes/macrophages. **(A)-(E)** Absolute number of cells per spleen. Data represent means ± SD of 3 to 5 animals per group of one experiment representative of two. **(F)-(J)** Fold modification of cell numbers in the infected mice in relation to naïve. Boxes and whiskers with 5–95 percentile and mean (showed by “+”) of 3 to 5 animals. Dashed and solid lines indicate a 2- or 4-fold modification, respectively, relative to naïve mice. One-way ANOVA followed by Dunnett’s multiple comparison test was performed to calculate statistically significant differences between naive and infected mice at 2 and 6 weeks after infection.

Along with the differences described in the parasite load, we analyzed the weight of spleen and liver of the infected animals and compared them with naïve mice (Additional file 6: Figure S5A and B). In general, small fluctuations were detected in the weight of the organs, which followed the trends exposed above for the parasite loads. Indeed, HL infected mice presented hepatosplenomegaly in the chronic phase of the disease, the pathologic hallmark of visceral leishmaniasis (reviewed in [44]). We attributed the splenomegaly not only to the presence of elevated numbers of *Leishmania* but also to the expansion of the main cellular populations detected in the spleen (Figure 6). A significant increase in both CD4^+^ and CD8^+^ T cells, B cells and macrophages was quantified in mice infected for 6 weeks with HL, an increment that corresponded to double the numbers found in the naïve mice. While the expansion of lymphocyte populations are classically linked to cellular and humoral immune responses, the presence and increase of CD11b^+^Ly6C^+^ monocytes were recently described to play a major role in the architectural remodeling of the spleen during experimental visceral leishmaniasis, mainly in the vascularization of the red pulp that accompanies splenomegaly [45].

**Figure 7:**
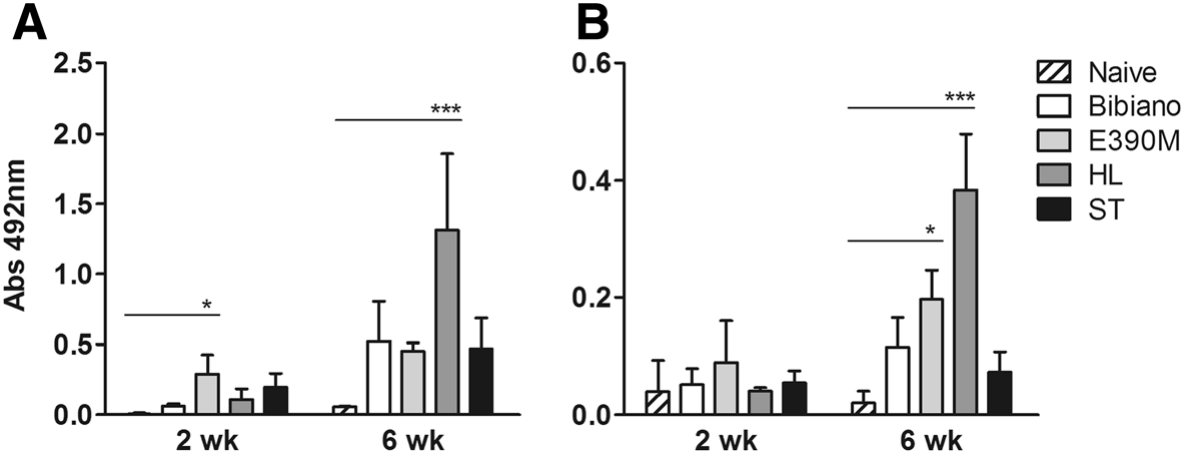
***Leishmania*-specific humoral response.***Leishmania*-specific sera reactivity of naive and infected animals 2 and 6 weeks post-infection was analyzed by ELISA. Specific **(A)** IgG2a and **(B)** IgG1 were quantified and are depicted as means ± SD of one representative experiment of 2 independents. Statistically significant differences are pointed out as given by one-way ANOVA followed by Dunnett’s multiple comparison test.

The enlargement in the B cell population explains the elevated titers of anti-*Leishmania* antibodies quantified in the chronic infection by HL (Figure 7). Both IgG2a and IgG1 were generated in high levels, leading to the exacerbation of the pathology, as described by others [46-48]. However, in E390M infections we did not detect any B cell expansion, as measured at 2 weeks post infection and later at 6 weeks, though IgG2a and IgG1 were significantly increased compared to age-matched naïve mice. We speculate that this antibody production could be in part related to the *k26* gene. *Leishmania* k26 (also known as HASPB) protein, as well as SHERP that share the same locus on chromosome 23 [49], are stage-regulated proteins, expressed only in the mammalian host infective forms (metacyclic promastigotes and amastigotes) [50]. k26 has a central core composed of a 10–11 amino acid repeats (PKEDGHTQKND/PKEDGRTQKN in Additional file 3: Figure S2) which present high inter and intra-specific variability [49,51,52], a feature that makes it an interesting tool for molecular typing of different *Leishmania* species and strains [21]. Other than this, k26 is a highly immunogenic antigen [49] with proven efficacy as a vaccine in murine models of visceral leishmaniasis by *L. donovani*[51,53] and partial efficacy in canine leishmaniasis by *L. infantum*[54]. Its immunogenic properties make k26 an interesting antigen that has been studied for diagnostic purposes [55-57]. As we reported in this work, E390M k26 protein has more repeats than MON-1 strains, which may influence the type and the strength of the humoral response as those amino acid repeats were determined to be B cell epitopes [52]. We point towards this argument since Bibiano and ST strains, that proved to be more infective than E390M, were not able to produce specific antibodies nor increased splenic cellularity in the acute or the chronic phases of murine VL.

## Conclusions

The molecular typing strategy confirmed the previous zymodeme characterization by MLEE and provided further knowledge that can be applied to diagnostics and population genetics studies. In this sense, the *k26* sequence for E390M strain generated in this work adds information for a zymodeme (MON-284) not included in the study by Harambolous [21], thus contributing to k*26* gene-based typing methodology, which is being used increasingly in population genetics and molecular epidemiology studies related to the *L. donovani* complex [58,59]. Laboratory conditions for *in vitro* culture were set to produce Bibiano, E390M, HL and ST fit promastigotes in the same developmental stage. *In vivo* infections with HL confirmed the *in vitro* phenotype of the most infective strain as more parasites were estimated to be present in the whole animal. ST was also considered to be highly infective though with a slower progression over time. Bibiano and E390M, isolated from HIV+ patients, showed differential infectivity and immunomodulation that could be influenced by the initial compartmentalization in host tissues. Interestingly, the most (HL) and the least (E390M) infective strains were the most immunogenic, revealing high levels of anti-*Leishmania* IgG2a and IgG1, especially in the chronic phase of infection.

This work is in line with previous studies [6,7,17,60] that show that leishmaniasis is a multifactorial disease and the broad spectrum of clinical manifestations depends on the genetics and inherent characteristics of the parasite coordinated with the susceptibility of the host.

## Competing interests

The authors declare no competing interest.

## Authors’ contributions

JC designed and performed all the experiments, analyzed data and wrote the manuscript. EC participated in some of the *in vivo* experiments, gave valuable input concerning qPCR for evaluation of the parasite load and helped to draft the manuscript. CS participated in the *in vivo* experiments, especially in collecting animal organs, and performed DNA extractions and qPCRs. IC carried out the molecular typing. JM and ACS conceived the study, participated in its design and coordination and helped to draft the manuscript. All authors read, discussed and approved the final manuscript.

## Supplementary Material

Additional file 1 Additional methods. Click here for file

Additional file 2: Figure S2 Validation of the qPCR methodology for quantification of the parasite loads in murine tissues. BALB/c mice were infected for 2 weeks with 10^8^ *L. infantum* strains (3 mice for each strain) cultivated in an equivolumetric mixture of RPMI and Schneider for 5 days. (**A**) Spleen and (**B**) liver were collected and cell suspensions were prepared. Parasite loads were quantified by qPCR and compared with the values obtained by limiting dilution assay. Click here for file

Additional file 3: Figure S2 *k26* gene alignment. Coding DNA sequences (cds) were translated into amino acids and aligned with standard sequences generated by Haralambous *et al.*[21] [GenBank: EF504255 and EF504256]. Complete cds of *k26* gene [GenBank: XM_001465758.2] is aligned in the first row. The four *L. infantum* isolates are aligned in the bottom rows. E390M *k26* sequence was entered in the GenBank with the access number KC576808. Click here for file

Additional file 4: Figure S3 Cell cycle analysis. (**A**) Representative histograms of PI content at day 1 and 6 of promastigote culture. (**B**-**D**) The percentage of (**B**) Bibiano, (**C**) E390M and (**D**) ST promastigotes in G1, S or G2 phases of the cell cycle was determined at days 1 to 6 of culture in NNN medium by flow cytometry after staining the parasites with a PI solution. Three independent experiments were performed, one representative experiment is shown. Click here for file

Additional file 5: Figure S4 Variation of the transcription of metacyclogenesis-dependent genes. *Meta*-1, *SHERP* and *histone H4* transcription was quantified by RT-PCR over the time of culture in NNN medium. To evaluate the progression of metacyclogenesis we related the up-regulation of (**A**) *Meta-1* and (**B**) *SHERP* with the down-regulation of *histone H4* applying a mathematical ratio. Bars represent the mean fold change relative to day 1 with SD of two independent experiments. Statistically significant differences between day 1 and the following days were determined with One-way ANOVA and Dunnett’s multiple comparison test. Click here for file

Additional file 6: Figure S5 Organ weight 2 and 6 weeks postinfection. Mice were sacrificed and (**A**) spleen, (**B**) liver and (**C**) inguinal lymph nodes were collected and weighted. Data represent means ± SD of 3¬5 animals of one experiment representative of two. One-way ANOVA followed by Dunnett’s multiple comparison test were run for statistical analysis between groups in each time point. Click here for file

## References

[B1] Chappuis F Sundar S Hailu A Ghalib H Rijal S Peeling RW Alvar J Boelaert M Visceral leishmaniasis: what are the needs for diagnosis, treatment and control? Nat Rev Microbiol 2007 5 11 873 882 17938629 10.1038/nrmicro1748

[B2] van Griensven J Diro E Visceral leishmaniasis Infect Dis Clin N Am 2012 26 2 309 322 10.1016/j.idc.2012.03.005 22632641

[B3] Goto H Lauletta Lindoso JA Cutaneous and mucocutaneous leishmaniasis Infect Dis Clin N Am 2012 26 2 293 307 10.1016/j.idc.2012.03.001 22632640

[B4] Alvar J Aparicio P Aseffa A Den Boer M Canavate C Dedet JP Gradoni L Ter Horst R Lopez-Velez R Moreno J The relationship between leishmaniasis and AIDS: the second 10 years Clini Microbiol Rev 2008 21 2 334 359 table of contents 10.1128/CMR.00061-07 PMC2292576 18400800

[B5] WHO Control of the leishmaniasis: report of a meeting of the WHO Expert Committee on the Control of Leishmaniases, Geneva, 22–26 March 2010 WHO Tech Rep Ser 2010 949 xii xiii 1–186, back cover 21485694

[B6] Maia C Seblova V Sadlova J Votypka J Volf P Experimental transmission of Leishmania infantum by two major vectors: a comparison between a viscerotropic and a dermotropic strain PLOS Neglect Trop D 2011 5 6 e1181 10.1371/journal.pntd.0001181 PMC3114756 21695108

[B7] Baptista-Fernandes T Marques C Roos Rodrigues O Santos-Gomes GM Intra-specific variability of virulence in Leishmania infantum zymodeme MON-1 strains Comp Immunol Microb 2007 30 1 41 53 10.1016/j.cimid.2006.10.001 17109961

[B8] Dey T Afrin F Anam K Ali N Infectivity and virulence of Leishmania donovani promastigotes: a role for media, source, and strain of parasite J Euk Microbiol 2002 49 4 270 274 10.1111/j.1550-7408.2002.tb00369.x 12188216

[B9] Moreira D Santarem N Loureiro I Tavares J Silva AM Amorim AM Ouaissi A Cordeiro-da-Silva A Silvestre R Impact of continuous axenic cultivation in Leishmania infantum virulence PLOS Neglect Trop D 2012 6 1 e1469 10.1371/journal.pntd.0001469 PMC3265455 22292094

[B10] Gradoni L Gramiccia M Leishmania infantum tropism: strain genotype or host immune status? Parasitol Today 1994 10 7 264 267 15275441 10.1016/0169-4758(94)90142-2

[B11] Laskay T Diefenbach A Rollinghoff M Solbach W Early parasite containment is decisive for resistance to Leishmania major infection Eur J Immunol 1995 25 8 2220 2227 10.1002/eji.1830250816 7664785

[B12] Gangneux JP Sulahian A Honore S Meneceur P Derouin F Garin YJ Evidence for determining parasitic factors in addition to host genetics and immune status in the outcome of murine Leishmania infantum visceral leishmaniasis Parasite Immunol 2000 22 10 515 519 10.1046/j.1365-3024.2000.00332.x 11012977

[B13] Chicharro C Jimenez MI Alvar J Iso-enzymatic variability of Leishmania infantum in Spain Ann Trop Med Parasit 2003 97 Suppl 1 57 64 14678633 10.1179/000349803225002534

[B14] Rivas L Moreno J Canavate C Alvar J Virulence and disease in leishmaniasis: what is relevant for the patient? Trends Parasitol 2004 20 7 297 301 10.1016/j.pt.2004.05.005 15193556

[B15] Pratlong F Dereure J Deniau M Marty P Faraut-Gambarelli F Dedet JP Enzymatic polymorphism during Leishmania/HIV co-infection: a study of 381 Leishmania strains received between 1986 and 2000 at the international cryobank in Montpellier, France Ann Trop Med Parasit 2003 97 Suppl 1 47 56 14678632 10.1179/000349803225002525

[B16] Gramiccia M Gradoni L Troiani M HIV-Leishmania co-infections in Italy. Isoenzyme characterization of Leishmania causing visceral leishmaniasis in HIV patients T Roy Soc Trop Med H 1992 86 2 161 163 10.1016/0035-9203(92)90551-M 1440776

[B17] Sulahian A Garin YJ Pratlong F Dedet JP Derouin F Experimental pathogenicity of viscerotropic and dermotropic isolates of Leishmania infantum from immunocompromised and immunocompetent patients in a murine model FEMS Immunol Med Mic 1997 17 3 131 138 10.1111/j.1574-695X.1997.tb01005.x 9093833

[B18] Silvestre R Cordeiro-Da-Silva A Santarem N Vergnes B Sereno D Ouaissi A SIR2-deficient Leishmania infantum induces a defined IFN-gamma/IL-10 pattern that correlates with protection J Immunol 2007 179 5 3161 3170 17709531 10.4049/jimmunol.179.5.3161

[B19] Fraga J Montalvo AM De Doncker S Dujardin JC Van der Auwera G Phylogeny of Leishmania species based on the heat-shock protein 70 gene Infect Genet Evol 2010 10 2 238 245 10.1016/j.meegid.2009.11.007 19913110

[B20] Kuhls K Mauricio IL Pratlong F Presber W Schonian G Analysis of ribosomal DNA internal transcribed spacer sequences of the Leishmania donovani complex Microbes Infect 2005 7 11–12 1224 1234 16002315 10.1016/j.micinf.2005.04.009

[B21] Haralambous C Antoniou M Pratlong F Dedet JP Soteriadou K Development of a molecular assay specific for the Leishmania donovani complex that discriminates L. donovani/Leishmania infantum zymodemes: a useful tool for typing MON-1 Diagn Micr Infec Dis 2008 60 1 33 42 10.1016/j.diagmicrobio.2007.07.019 17889482

[B22] Hall TA BioEdit: a user-friendly biological sequence alignment editor and analysis program for Windows 95/98/NT Nucl Acid S 1999 41 95 98

[B23] Inocencio da Luz R Romero GA Dorval ME Cruz I Canavate C Dujardin JC Van Assche T Cos P Maes L Drug susceptibility of Leishmania infantum (syn. Leishmania chagasi) isolates from Brazilian HIV-positive and HIV-negative patients J Antimicrob Chemoth 2011 66 3 677 679 10.1093/jac/dkq508 21393233

[B24] Adaui V Schnorbusch K Zimic M Gutierrez A Decuypere S Vanaerschot M De Doncker S Maes I Llanos-Cuentas A Chappuis F Comparison of gene expression patterns among Leishmania braziliensis clinical isolates showing a different in vitro susceptibility to pentavalent antimony Parasitology 2011 138 2 183 193 10.1017/S0031182010001095 20678296

[B25] Ouakad M Bahi-Jaber N Chenik M Dellagi K Louzir H Selection of endogenous reference genes for gene expression analysis in Leishmania major developmental stages Parasitology Res 2007 101 2 473 477 10.1007/s00436-007-0491-1 17318579

[B26] van Eys GJ Schoone GJ Kroon NC Ebeling SB Sequence analysis of small subunit ribosomal RNA genes and its use for detection and identification of Leishmania parasites Mol Biochem Parasit 1992 51 1 133 142 10.1016/0166-6851(92)90208-2 1565128

[B27] Miro G Oliva G Cruz I Canavate C Mortarino M Vischer C Bianciardi P Multicentric, controlled clinical study to evaluate effectiveness and safety of miltefosine and allopurinol for canine leishmaniosis Vet Dermatol 2009 20 5–6 397 404 20178476 10.1111/j.1365-3164.2009.00824.x

[B28] Cruz I Chicharro C Nieto J Bailo B Canavate C Figueras MC Alvar J Comparison of new diagnostic tools for management of pediatric Mediterranean visceral leishmaniasis J Clin Microbiol 2006 44 7 2343 2347 10.1128/JCM.02297-05 16825347 PMC1489479

[B29] Mahmoudzadeh-Niknam H Abrishami F Doroudian M Moradi M Alimohammadian M Parvizi P Hatam G Mohebali M Khalaj V The Problem of Mixing up of Leishmania Isolates in the Laboratory: Suggestion of ITS1 Gene Sequencing for Verification of Species Iran J Parasitol 2011 6 1 41 48 22347273 PMC3279869

[B30] Simpson L Holz G Jr The status of Leishmania tarentolae/Trypanosoma platydactyli Parasitol Today 1988 4 4 115 118 10.1016/0169-4758(88)90043-9 15463063

[B31] Neal RA Leishmania major: culture media, mouse strains, and promastigote virulence and infectivity Exp Parasitol 1984 57 3 269 273 10.1016/0014-4894(84)90100-0 6723897

[B32] Kebaier C Louzir H Chenik M Ben Salah A Dellagi K Heterogeneity of wild Leishmania major isolates in experimental murine pathogenicity and specific immune response Infect Immun 2001 69 8 4906 4915 10.1128/IAI.69.8.4906-4915.2001 11447167 PMC98581

[B33] Santos MG Silva MF Zampieri RA Lafraia RM Floeter-Winter LM Correlation of meta 1 expression with culture stage, cell morphology and infectivity in Leishmania (Leishmania) amazonensis promastigotes Mem Inst Oswaldo Cruz 2011 106 2 190 193 21537679 10.1590/s0074-02762011000200012

[B34] Saraiva EM Pinto-da-Silva LH Wanderley JL Bonomo AC Barcinski MA Moreira ME Flow cytometric assessment of Leishmania spp metacyclic differentiation: validation by morphological features and specific markers Exp Parasitol 2005 110 1 39 47 10.1016/j.exppara.2005.01.004 15804377

[B35] Soto M Quijada L Alonso C Requena JM Molecular cloning and analysis of expression of the Leishmania infantum histone H4 genes Mol Biochem Parasitol 1997 90 2 439 447 10.1016/S0166-6851(97)00178-3 9476792

[B36] Cortez M Huynh C Fernandes MC Kennedy KA Aderem A Andrews NW Leishmania promotes its own virulence by inducing expression of the host immune inhibitory ligand CD200 Cell Host Microbe 2011 9 6 463 471 10.1016/j.chom.2011.04.014 21669395 PMC3118640

[B37] Gomes IN Calabrich AF Tavares Rda S Wietzerbin J de Freitas LA Veras PS Differential properties of CBA/J mononuclear phagocytes recovered from an inflammatory site and probed with two different species of Leishmania Microbes Infect 2003 5 4 251 260 10.1016/S1286-4579(03)00025-X 12706438

[B38] Wilson ME Jeronimo SM Pearson RD Immunopathogenesis of infection with the visceralizing Leishmania species Microb Pathogenesis 2005 38 4 147 160 10.1016/j.micpath.2004.11.002 15797810

[B39] Sacks D Noben-Trauth N The immunology of susceptibility and resistance to Leishmania major in mice Nat Rev Immunol 2002 2 11 845 858 10.1038/nri933 12415308

[B40] Colvin GA Lambert JF Abedi M Hsieh CC Carlson JE Stewart FM Quesenberry PJ Murine marrow cellularity and the concept of stem cell competition: geographic and quantitative determinants in stem cell biology Leukemia 2004 18 3 575 583 10.1038/sj.leu.2403268 14749701

[B41] Diehl KH Hull R Morton D Pfister R Rabemampianina Y Smith D Vidal JM van de Vorstenbosch C European Federation of Pharmaceutical Industries A, European Centre for the Validation of Alternative M A good practice guide to the administration of substances and removal of blood, including routes and volumes J Appl Toxicol 2001 21 1 15 23 10.1002/jat.727 11180276

[B42] Cruz I Morales MA Noguer I Rodriguez A Alvar J Leishmania in discarded syringes from intravenous drug users Lancet 2002 359 9312 1124 1125 10.1016/S0140-6736(02)08160-6 11943264

[B43] Cruz I Nieto J Moreno J Canavate C Desjeux P Alvar J Leishmania/HIV co-infections in the second decade Indian J Med Res 2006 123 3 357 388 16778317

[B44] Stanley AC Engwerda CR Balancing immunity and pathology in visceral leishmaniasis Immunol Cell Biol 2007 85 2 138 147 10.1038/sj.icb7100011 17146466

[B45] Yurdakul P Dalton J Beattie L Brown N Erguven S Maroof A Kaye PM Compartment-specific remodeling of splenic micro-architecture during experimental visceral leishmaniasis American J Pathol 2011 179 1 23 29 10.1016/j.ajpath.2011.03.009 PMC3123882 21703391

[B46] Oliveira DM Costa MA Chavez-Fumagalli MA Valadares DG Duarte MC Costa LE Martins VT Gomes RF Melo MN Soto M Evaluation of parasitological and immunological parameters of Leishmania chagasi infection in BALB/c mice using different doses and routes of inoculation of parasites Parasitol Res 2012 110 3 1277 1285 10.1007/s00436-011-2628-5 21915627

[B47] Deak E Jayakumar A Cho KW Goldsmith-Pestana K Dondji B Lambris JD McMahon-Pratt D Murine visceral leishmaniasis: IgM and polyclonal B-cell activation lead to disease exacerbation Eur J Immunol 2010 40 5 1355 1368 10.1002/eji.200939455 20213734 PMC2944234

[B48] Kaur S Kaur T Garg N Mukherjee S Raina P Athokpam V Effect of dose and route of inoculation on the generation of CD4+ Th1/Th2 type of immune response in murine visceral leishmaniasis Parasitol Res 2008 103 6 1413 1419 10.1007/s00436-008-1150-x 18751727

[B49] Depledge DP MacLean LM Hodgkinson MR Smith BA Jackson AP Ma S Uliana SR Smith DF Leishmania-specific surface antigens show sub-genus sequence variation and immune recognition PLOS Neglect Trop D 2010 4 9 e829 10.1371/journal.pntd.0000829 PMC2946902 20927190

[B50] Sadlova J Price HP Smith BA Votypka J Volf P Smith DF The stage-regulated HASPB and SHERP proteins are essential for differentiation of the protozoan parasite Leishmania major in its sand fly vector, Phlebotomus papatasi Cell Microbiol 2010 12 12 1765 1779 10.1111/j.1462-5822.2010.01507.x 20636473 PMC3015063

[B51] Maroof A Brown N Smith B Hodgkinson MR Maxwell A Losch FO Fritz U Walden P Lacey CN Smith DF Therapeutic vaccination with recombinant adenovirus reduces splenic parasite burden in experimental visceral leishmaniasis J Infect Dis 2012 205 5 853 863 10.1093/infdis/jir842 22301630 PMC3274377

[B52] Zackay A Nasereddin A Takele Y Tadesse D Hailu W Hurissa Z Yifru S Weldegebreal T Diro E Kassahun A Polymorphism in the HASPB Repeat Region of East African Leishmania donovani Strains PLOS Neglect Trop D 2013 7 1 e2031 10.1371/journal.pntd.0002031 PMC3554577 23358849

[B53] Stager S Smith DF Kaye PM Immunization with a recombinant stage-regulated surface protein from Leishmania donovani induces protection against visceral leishmaniasis J Immunol 2000 165 12 7064 7071 11120835 10.4049/jimmunol.165.12.7064

[B54] Moreno J Nieto J Masina S Canavate C Cruz I Chicharro C Carrillo E Napp S Reymond C Kaye PM Immunization with H1, HASPB1 and MML Leishmania proteins in a vaccine trial against experimental canine leishmaniasis Vaccine 2007 25 29 5290 5300 10.1016/j.vaccine.2007.05.010 17576026 PMC2695600

[B55] Farajnia S Darbani B Babaei H Alimohammadian MH Mahboudi F Gavgani AM Development and evaluation of Leishmania infantum rK26 ELISA for serodiagnosis of visceral leishmaniasis in Iran Parasitology 2008 135 9 1035 1041 18561868 10.1017/S003118200800454X

[B56] Sundar S Singh RK Bimal SK Gidwani K Mishra A Maurya R Singh SK Manandhar KD Boelaert M Rai M Comparative evaluation of parasitology and serological tests in the diagnosis of visceral leishmaniasis in India: a phase III diagnostic accuracy study Trop Med Int Health: TM & IH 2007 12 2 284 289 17300637 10.1111/j.1365-3156.2006.01775.x

[B57] da Costa RT Franca JC Mayrink W Nascimento E Genaro O Campos-Neto A Standardization of a rapid immunochromatographic test with the recombinant antigens K39 and K26 for the diagnosis of canine visceral leishmaniasis T Roy Soc Trop Med H 2003 97 6 678 682 10.1016/S0035-9203(03)80102-5 16117962

[B58] Bhattarai NR Dujardin JC Rijal S De Doncker S Boelaert M Van der Auwera G Development and evaluation of different PCR-based typing methods for discrimination of Leishmania donovani isolates from Nepal Parasitology 2010 137 6 947 957 10.1017/S0031182009991752 20109247

[B59] Gouzelou E Haralambous C Amro A Mentis A Pratlong F Dedet JP Votypka J Volf P Toz SO Kuhls K Multilocus microsatellite typing (MLMT) of strains from Turkey and Cyprus reveals a novel monophyletic L. donovani sensu lato group PLOS Neglect Trop D 2012 6 2 e1507 10.1371/journal.pntd.0001507 PMC3279343 22348162

[B60] Wege AK Florian C Ernst W Zimara N Schleicher U Hanses F Schmid M Ritter U Leishmania major infection in humanized mice induces systemic infection and provokes a nonprotective human immune response PLOS Neglect Trop D 2012 6 7 e1741 10.1371/journal.pntd.0001741 PMC3404120 22848771

