# Supplementary material for: Characterization of the biology and infectivity of Leishmania infantum viscerotropic and dermotropic strains isolated from HIV+ and HIV- patients in the murine model of visceral leishmaniasis
Source: Parasit Vectors. 2013 Apr 26;6:122. doi: 10.1186/1756-3305-6-122 (PMC3649922; doi:10.1186/1756-3305-6-122)
Supplement: Additional file 1 — Additional methods. [file 1756-3305-6-122-S1.doc]

**ADDITIONAL METHODS**

**Culture media referenced in unpublished data**

RPMI 20% FBS + glucose medium was produced by enrichment of supplemented RPMI with an extra 10% of FBS and 2.5 mg/mL of glucose (Sigma-Aldrich). Schneider is composed of Schneider’s Insect Medium (Sigma-Aldrich) supplemented with 10% FBS, 200 units/mL penicillin, 200 units/mL streptomycin, 5 mM Hepes Buffer, 2.5 g/mL Phenol Red (Sigma-Aldrich). A mixture of Schneider and RPMI in equal parts (50% SchRPMI) was also used. Promastigotes were cultivated at 26 ºC with an initial inoculum of 10^6^ parasites /mL (or 10^5^ parasites/mL in Schneider) from a synchronized culture in the same media and followed for 6 days.

**Parasite load quantification by limiting dilution**

Spleen and liver of each animal were collected and homogenized into cell suspensions in

50% SchRPMI medium. A fraction of the total organ was plated in a 96-well plate and submitted to 2-fold serial dilutions in the same culture medium. After 2 weeks of incubation at 26 ºC, the quantification of the parasite load was carried out as described in [18] by the integration of the dilution factor of the last well where at least one parasite was detected with the initial amount of organ plated on the first well and its total weight.
