# Supplementary figures and images for: Characterization of the biology and infectivity of Leishmania infantum viscerotropic and dermotropic strains isolated from HIV+ and HIV- patients in the murine model of visceral leishmaniasis
Source: Parasit Vectors. 2013 Apr 26;6:122. doi: 10.1186/1756-3305-6-122 (PMC3649922; doi:10.1186/1756-3305-6-122)

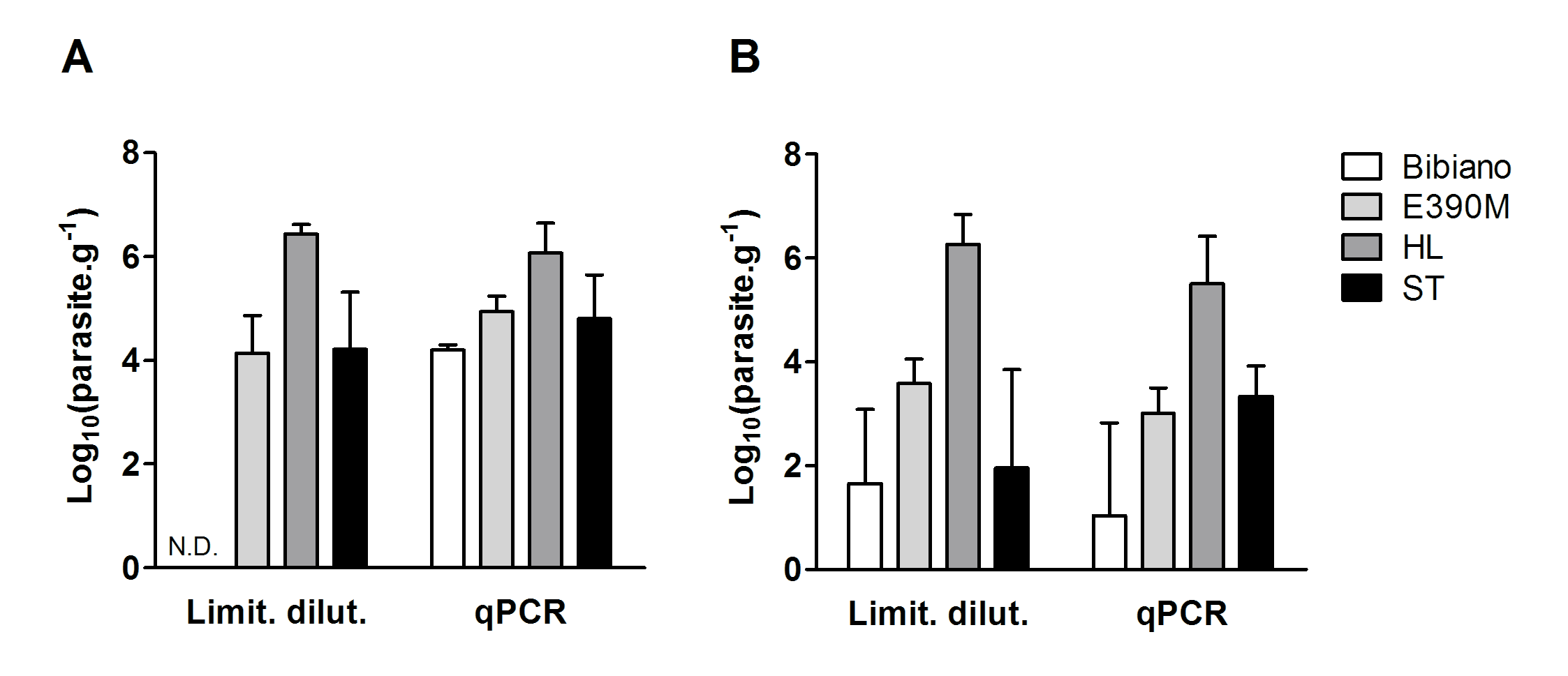

Supplement: Additional file 2: Figure S2 — Validation of the qPCR methodology for quantification of the parasite loads in murine tissues. BALB/c mice were infected for 2 weeks with 108 L. infantum strains (3 mice for each strain) cultivated in an equivolumetric mixture of RPMI and Schneider for 5 days. (A) Spleen and (B) liver were collected and cell suspensions were prepared. Parasite loads were quantified by qPCR and compared with the values obtained by limiting dilution assay. [file 1756-3305-6-122-S2.tiff]

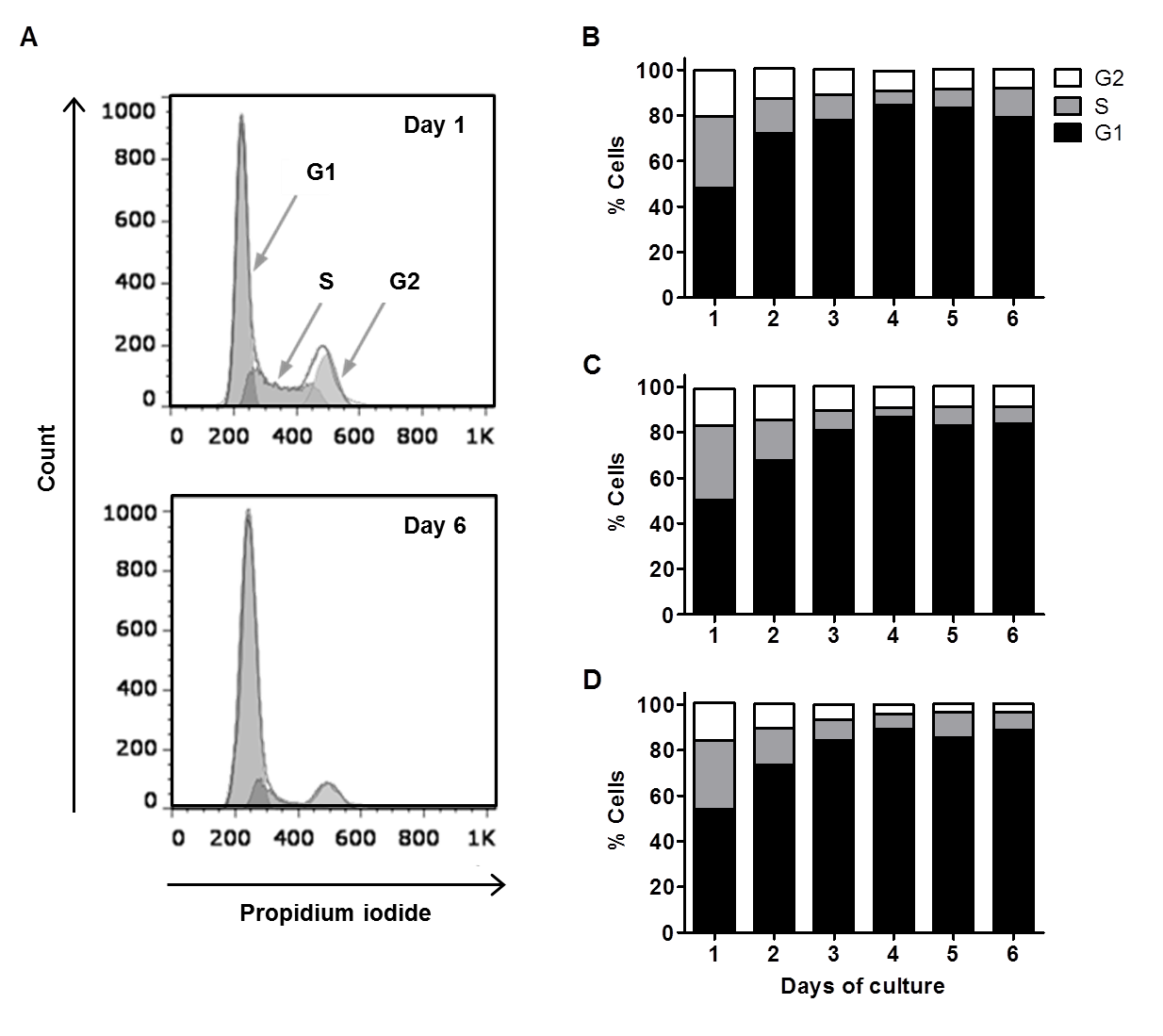

Supplement: Additional file 4: Figure S3 — Cell cycle analysis. (A) Representative histograms of PI content at day 1 and 6 of promastigote culture. (B-D) The percentage of (B) Bibiano, (C) E390M and (D) ST promastigotes in G1, S or G2 phases of the cell cycle was determined at days 1 to 6 of culture in NNN medium by flow cytometry after staining the parasites with a PI solution. Three independent experiments were performed, one representative experiment is shown. [file 1756-3305-6-122-S4.tiff]

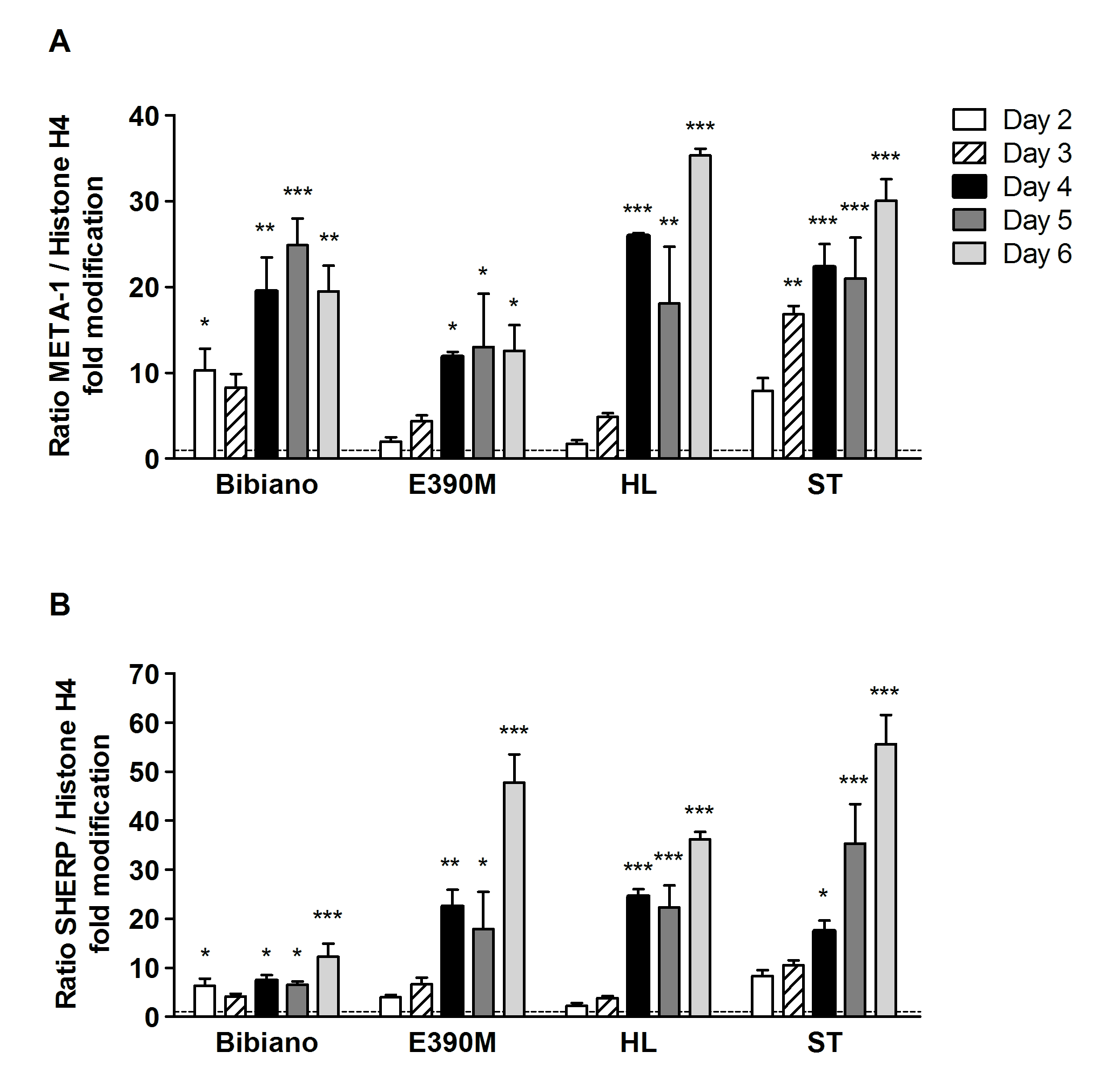

Supplement: Additional file 5: Figure S4 — Variation of the transcription of metacyclogenesis-dependent genes. Meta-1, SHERP and histone H4 transcription was quantified by RT-PCR over the time of culture in NNN medium. To evaluate the progression of metacyclogenesis we related the up-regulation of (A) Meta-1 and (B) SHERP with the down-regulation of histone H4 applying a mathematical ratio. Bars represent the mean fold change relative to day 1 with SD of two independent experiments. Statistically significant differences between day 1 and the following days were determined with One-way ANOVA and Dunnett’s multiple comparison test. [file 1756-3305-6-122-S5.tiff]

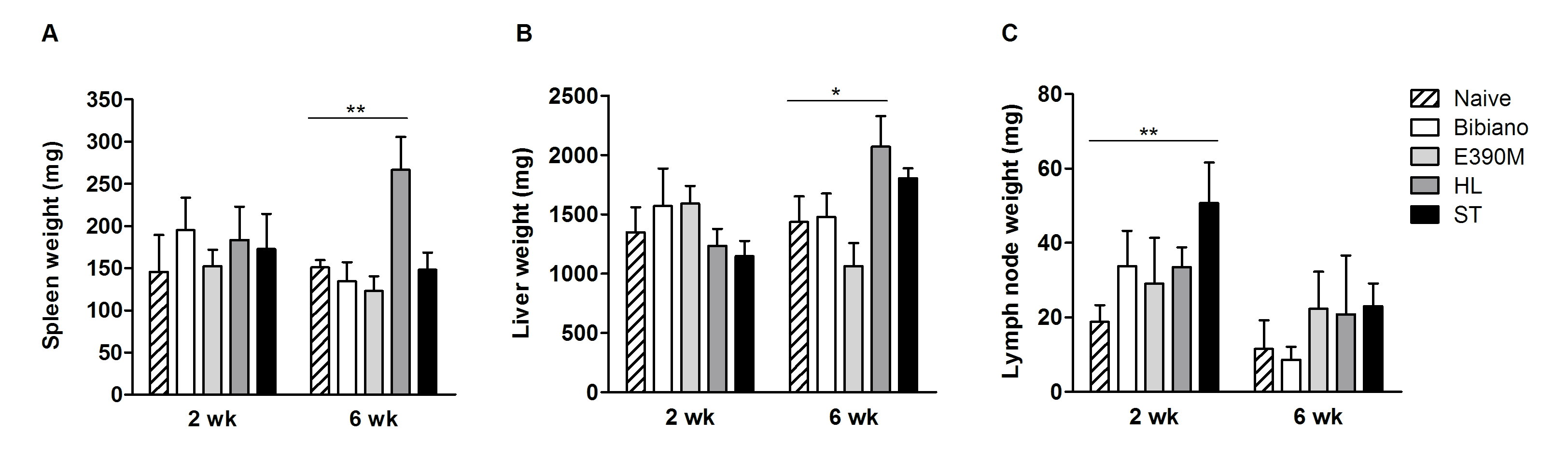

Supplement: Additional file 6: Figure S5 — Organ weight 2 and 6 weeks postinfection. Mice were sacrificed and (A) spleen, (B) liver and (C) inguinal lymph nodes were collected and weighted. Data represent means ± SD of 3¬5 animals of one experiment representative of two. One-way ANOVA followed by Dunnett’s multiple comparison test were run for statistical analysis between groups in each time point. [file 1756-3305-6-122-S6.tiff]
