# Supplementary material for: Characterization of the biology and infectivity of Leishmania infantum viscerotropic and dermotropic strains isolated from HIV+ and HIV- patients in the murine model of visceral leishmaniasis
Source: Parasit Vectors. 2013 Apr 26;6:122. doi: 10.1186/1756-3305-6-122 (PMC3649922; doi:10.1186/1756-3305-6-122)
Supplement: Additional file 3: Figure S2 — k26 gene alignment. Coding DNA sequences (cds) were translated into amino acids and aligned with standard sequences generated by Haralambous et al.[21] [GenBank: EF504255 and EF504256]. Complete cds of k26 gene [GenBank: XM_001465758.2] is aligned in the first row. The four L. infantum isolates are aligned in the bottom rows. E390M k26 sequence was entered in the GenBank with the access number KC576808. [file 1756-3305-6-122-S3.pdf]

```
                    ....|....| ....|....| ....|....| ....|....| ....|....|
                            10         20         30         40         50
XM_001465758.2      MGAYCTKDSA KEPQKRADNI HKTTEANHRG AAGVPPKHAG GAMNDSAPKE
EF504255            ---------- ------ADNI HKTTEANHRG AAGVPPKHAG GAMNDSAPKE
EF504256            ---------- ------ADNI HKTTEANHRG AAGVPPKHAG GAMNDSAPKE
BIBIANO_K26         -----TKDSA KEPQKRADNI HKTTEANHRG AAGVPPKHAG GAMNDSAPKE
E390M_K26           -----TKDSA KEPQKRADNI HKTTEANHRG AAGVPPKHAG GAMNDSAPKE
HL_K26              -----TKDSA KEPQKRADNI HKTTEANHRG AAGVPPKHAG GAMNDSAPKE
ST_K26              -----TKDSA KEPQKRADNI HKTTEANHRG AAGVPPKHAG GAMNDSAPKE

                    ....|....| ....|....| ....|....| ....|....| ....|....|
                            60         70         80         90        100
XM_001465758.2      DGHTQKNDGD GPKEDGRTQK NDDGGPKEDG HTQKNDGDGP KEDGRTQKNN
EF504255            DGHTQKNDGD GPKEDGHTQK NDDGGPKEDG HTQKNDGDGP KEDGRTQKNN
EF504256            DGHTQKNDGD GPKEDGRTQK NDDGGPKEDG HTQKNDGDGP KEDGRTQKNN
BIBIANO_K26         DGHTQKNDGD GPKEDGRTQK NDDGGPKEDG HTQKNDGDGP KEDGRTQKNN
E390M_K26           DGHTQKNDGD GPKEDGHTQK NDDGGPKEDG HTQKNDGDGP KEDGRTQKND
HL_K26              DGHTQKNDGD GPKEDGRTQK NDDGGPKEDG HTQKNDGDGP KEDGRTQKNN
ST_K26              DGHTQKNDGD GPKEDGRTQK NDDGGPKEDG HTQKNDGDGP KEDGRTQKNN

                    ....|....| ....|....| ....|....| ....|....| ....|....|
                           110        120        130        140        150
XM_001465758.2      GDGPKEDGHT QKNDGDAPKE DGRTQKNDGD GPKEDGRTQK NDGDGPKEDG
EF504255            GDGPKEDGHT QKNDGDAPKE DGRTQKNDGD GPKEDGRTQK NDGDGPKEDG
EF504256            GDGPKEDGHT QKNDGDAPKE DGRTQKNDGD GPKEDGRTQK NDGDGPKEDG
BIBIANO_K26         GDGPKEDGHT QKNDGDAPKE DGRTQKNDGD GPKEDGRTQK NDGDGPKEDG
E390M_K26           GDGPKEDGRT QKNNGDGPKE DGRTQKNDGD GPKEDGRTQK NDGDGPKEDG
HL_K26              GDGPKEDGHT QKNDGDAPKE DGRTQKNDGD GPKEDGRTQK NDGDGPKEDG
ST_K26              GDGPKEDGHT QKNDGDAPKE DGRTQKNDGD GPKEDGRTQK NDGDGPKEDG

                    ....|....| ....|....| ....|....| ....|....| ....|....|
                           160        170        180        190        200
XM_001465758.2      RTQKNDGDGP KEDGRTQKND GDGPKEDGHT QKNDGDGPKE DGRTQKNDGG
EF504255            RTQKNDGDGP KEDGRTQKND GDGPKEDGHT QKNDGDGPKE DGRTQKNDGG
EF504256            RTQKNDGDGP KEDGRTQKND GDGPKEDGHT QKNDGDGPKE DGRTQKNDGG
BIBIANO_K26         RTQKNDGDGP KEDGRTQKND GDGPKEDGHT QKNDGDGPKE DGRTQKNDGG
E390M_K26           RTQKNDGDGP KEDGHTQKND GDGPKEDGHT QKNDGDGPKE DGRTQKNDGD
HL_K26              RTQKNDGDGP KEDGRTQKND GDGPKEDGHT QKNDGDGPKE DGRTQKNDGG
ST_K26              RTQKNDGDGP KEDGRTQKND GDGPKEDGHT QKNDGDGPKE DGRTQKNDGG

                    ....|....| ....|....| ....|....| ....|....| ....|....|
                           210        220        230        240        250
XM_001465758.2      GPKEDENLQQ NDGNAQEKNE DGHNVGDGAN GNEDGNDDQP KEQVAGN*..
EF504255            GPKE...... .......... .......... .......... ..........
EF504256            GPKE...... .......... .......... .......... ..........
BIBIANO_K26         GPKEDENLQQ NDG....... .......... .......... ..........
E390M_K26           GPKEDGHTQK NDGGGPKEDG RTQKNDGDGP KEDGHTQKND GGGPKEDGRT
HL_K26              GPKEDENLQQ NDG....... .......... .......... ..........
ST_K26              GPKEDENLQQ NDG....... .......... .......... ..........

                    ....|....| ....|....| ....|....| ...
                           260        270        280
XM_001465758.2      .......... .......... .......... ...
EF504255            .......... .......... .......... ...
EF504256            .......... .......... .......... ...
BIBIANO_K26         .......... .......... .......... ...
E390M_K26           QKNDGDGPKE DGRTQKNDGG GPKEDENLQQ NDG
HL_K26              .......... .......... .......... ...
ST_K26              .......... .......... .......... ...
```
